# Rift Valley Fever Potential, Arabian Peninsula

**DOI:** 10.3201/eid1203.050973

**Published:** 2006-03

**Authors:** Assaf Anyamba, Jean-Paul Chretien, Pierre B.H. Formenty, Jennifer Small, Compton J. Tucker, Joseph L. Malone, Hassan El Bushra, Vincent Martin, Kenneth J. Linthicum

**Affiliations:** * NASA/Goddard Space Flight Center, Greenbelt, Maryland, USA;; † Department of Defense Global Emerging Infections Surveillance and Response System, Silver Spring, Maryland, USA;; ‡ World Health Organization, Geneva, Switzerland;; § World Health Organization, Cairo, Egypt;; ¶ Food and Agricultural Organization, Rome, Italy;; # United States Department of Agriculture, Gainesville, Florida, USA

**Keywords:** pythiosis, arteritis, necrotizing cellulitis, letter

**To the Editor:** Rift Valley fever (RVF) virus causes severe disease, abortion, and death in domestic animals (especially young sheep, cattle, and goats) in Africa and the Arabian Peninsula. Humans are infected by mosquitoes, which maintain epizootic transmission, or through exposure to infected animal tissue. Although human disease may be mild, sometimes severe retinitis, meningoencephalitis, or hemorrhagic fever syndromes may develop in patients. In Africa, epizootics and associated human epidemics usually follow heavy rainfall (*1*).

RVF was first confirmed outside Africa in September 2000. The outbreak in southwestern coastal Saudi Arabia and neighboring coastal areas of Yemen resulted in an epizootic with >120 human deaths and major losses in livestock populations from disease and slaughter (*2**,**3*). RVF virus isolated from the floodwater mosquito *Aedes vexans arabiensis* during the outbreak was closely related to strains from Madagascar (1991) and Kenya (1997), which suggests that the virus was imported through infected mosquitoes or livestock from East Africa (*3*). The Arabian outbreak followed increased rainfall in nearby highlands that flooded the coastal areas and created ideal environments for mosquito populations similar to those found in RVF-endemic regions of East Africa (*4*). Most RVF activity was associated with flooded wadi agricultural systems; no cases were reported in the mountains or in dry sandy regions, where surface water does not accumulate long enough to sustain mosquito breeding.

**Figure A1:**
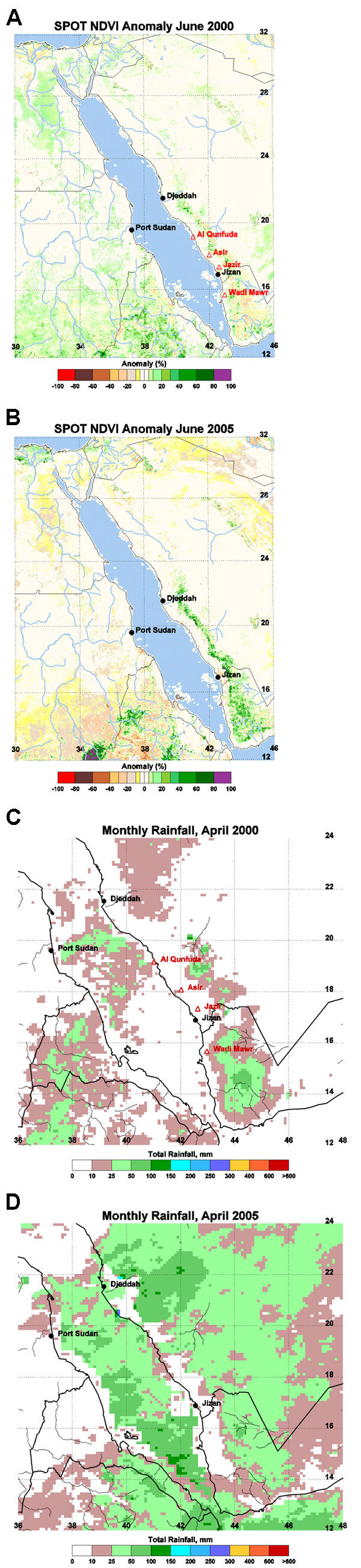
Figure A1. Systeme Probatoire pour l'Observation de la Terre vegetation sensor 1 km normalized difference vegetation index (NDVI) anomaly images of the Arabian Peninsula region during June 2000 (A) and June 2005 (B). Data are the percentage deviation from the long-term mean calculated for the period January 1999–June 2005 in NDVI units. A value of zero indicates that current values are identical to the 1998–2005 mean. Rift Valley fever virus was isolated in the Al Qunfuda, Asir, and Jazir areas in Saudi Arabia and in Wadi Mawr in Yemen between August and September 2000 (panel A). Positive anomalies in June 2005 are of higher magnitude than in June 2000. Satellite rainfall estimates were blended with rain-gauge data where available for April 2000 (C) and April 2005 (D). More widespread rainfall occurred in April 2005 compared with April 2000, which suggests prime conditions for vector breeding and prevalence in 2005.

To provide early warning of conditions favorable for RVF epidemics, the National Aeronautics and Space Administration (NASA) and the Department of Defense Global Emerging Infections Surveillance and Response System (DoD-GEIS) monitor the satellite-derived normalized difference vegetation index (NDVI), which reflects recent rainfall and other ecologic and climatic factors (*5**–**7*). NDVI anomalies in the highlands east of affected areas during the 2000 outbreak (Figure A1 panel A) showed a spatial pattern (although of lower magnitude) similar to recent anomalies in those areas (Figure A1 panel B). Greater than normal NDVIs (20%–60%) were seen in the Sarawat Mountains, from just northeast of Djeddah, Saudi Arabia, and southwestward beyond Jizan and into Hodeidah governorate in Yemen during May and June 2005.

Satellite-derived rainfall estimates show that widespread rainfall occurred over most of western Saudi Arabia and Yemen from mid-April to mid-June 2005 (*8*) and accounts for the high magnitude and spatial pattern of observed NDVI anomalies in May and June 2005. Rainfall was concentrated in the mountainous regions east of the Red Sea coast, and was heaviest in the areas east of Djeddah and Jizan, with rainfall totals as high as 120 mm and 60–80 mm, respectively, during April 2005, compared with the same period in 2000 (10–50 mm) (Figure A1 panels C and D) and in southwestern Yemen, with totals as high as 120 mm during May. In the area east of Djeddah, total rainfall in April 2005 was 150 mm above the long-term average for that month. Flooding was reported in Hodeidah Governorate, Yemen during May (*9*) and could be expected in other Red Sea coastal areas following such heavy rainfall. This created habitats appropriate for breeding of mosquitoes capable of transmitting RVF, as occurred in 2000.

No human cases of RVF have been reported in Saudi Arabia and Yemen since the 2000 outbreak, but in September 2004 the Saudi Ministry of Agriculture reported that 5 RVF-seropositive sheep had been identified during routine surveillance in Jizan where most infected persons were exposed during the outbreak in 2000 (*2*). The primary infection was estimated to have occurred in April 2004 (*10*). The NDVIs and rainfall patterns alerted the Yemen and Saudi Arabia Ministries of Health and Ministries of Agriculture to conduct field investigations with the Food and Agriculture Organization and the World Health Organization.

Since RVF virus can be maintained in mosquito eggs for extended periods and transmitted under favorable conditions (*6*), the high magnitude of NDVI and rainfall patterns reported should prompt heightened veterinary and human surveillance for RVF in coastal Arabia and mass vaccination of susceptible animals. The current RVF model (*7*) is indicative of conditions that would promote vector breeding and could result in an outbreak of mosquitoborne diseases.
